# Protein Interactions in *Xenopus* Germ Plasm RNP Particles

**DOI:** 10.1371/journal.pone.0080077

**Published:** 2013-11-12

**Authors:** Sarbjit Nijjar, Hugh R. Woodland

**Affiliations:** School of Life Sciences, University of Warwick, Coventry, Warwickshire, United Kingdom; CNRS UMR7275, France

## Abstract

Hermes is an RNA-binding protein that we have previously reported to be found in the ribonucleoprotein (RNP) particles of *Xenopus* germ plasm, where it is associated with various RNAs, including that encoding the germ line determinant Nanos1. To further define the composition of these RNPs, we performed a screen for Hermes-binding partners using the yeast two-hybrid system. We have identified and validated four proteins that interact with Hermes in germ plasm: two isoforms of Xvelo1 (a homologue of zebrafish Bucky ball) and Rbm24b and Rbm42b, both RNA-binding proteins containing the RRM motif. GFP-Xvelo fusion proteins and their endogenous counterparts, identified with antisera, were found to localize with Hermes in the germ plasm particles of large oocytes and eggs. Only the larger Xvelo isoform was naturally found in the Balbiani body of previtellogenic oocytes. Bimolecular fluorescence complementation (BiFC) experiments confirmed that Hermes and the Xvelo variants interact in germ plasm, as do Rbm24b and 42b. Depletion of the shorter Xvelo variant with antisense oligonucleotides caused a decrease in the size of germ plasm aggregates and loosening of associated mitochondria from these structures. This suggests that the short Xvelo variant, or less likely its RNA, has a role in organizing and maintaining the integrity of germ plasm in *Xenopus* oocytes. While GFP fusion proteins for Rbm24b and 42b did not localize into germ plasm as specifically as Hermes or Xvelo, BiFC analysis indicated that both interact with Hermes in germ plasm RNPs. They are very stable in the face of RNA depletion, but additive effects of combinations of antisense oligos suggest they may have a role in germ plasm structure and may influence the ability of Hermes protein to effectively enter RNP particles.

## Introduction

In *Xenopus*, as in many other organisms, the formation of the germline depends on germ plasm, a structure composed of aggregated particles, containing stored mRNAs and a variety of other components, notably mitochondria, anchored in the vegetal cortex. These are inherited by vegetal blastomeres during cleavage and direct them to become primordial germ cells (PGCs) [1]–[3]. Our previous data suggests that the RNAs are present in the same type of particle, but there is also a second kind of particle containing the protein Xpat [4]. One of the stored RNAs encodes a translational repressor protein, the *Xenopus* homologue of Nanos (in *Xenopus* now called Nanos1, but formerly called Xcat2) that is necessary to repress transcription in the blastomeres that inherit germ plasm during cleavage. This protects them from somatic differentiation forced by localised maternal molecules [5], [6].

As we have explained previously, there is a potential confusion about the word “granule” as applied to amphibian germ plasm. In older work the large aggregates found in eggs are called germinal granules, whereas in high resolution analyses “granule” may be used for the constituent particles of the aggregates. We therefore, follow some previous authors [7], [8] and refer to the aggregates as “islands”, containing constituent “particles”. It should also be mentioned that while some particles are RNPs, there is no evidence that others containing Xpat and Poc1B proteins contain RNA [4], [9], [10].

Hermes is the only protein so far reported to be present in the RNPs of mature *Xenopus* germ plasm [4], [11] Hermes is an RNA-binding protein containing a single RNA recognition motif (RRM) [12]. It is absent from germ plasm particles containing Xpat, and from the RNP particles containing axis-forming RNAs, like *VegT* and *Vg1*, localised by the so-called late pathway during vitellogenesis [4]. It should also be mentioned that in *Xenopus* only a fraction of Hermes protein is present in germ plasm, and immunoprecipitation studies reveal that it is associated with *RINGO/Spy* and *Mos* mRNAs, as well as *nanos1* (*Xcat2*) mRNA [11]. However, these are not necessarily in the same complexes. On an evolutionary note, Hermes protein has also been shown to be present in the germ plasm RNPs of the zebrafish [13].

In zebrafish Bucky ball (Buc) protein is another likely constituent of early germ plasm particles. Buc is essential for the formation of the Balbiani body (mitochondrial cloud) and it is needed to establish overall oocyte polarity [14], [15]. Moreover, over-expression of Buc leads to an increased number of PGCs, suggesting it has a crucial role in germline development, perhaps through organising germ plasm in the embryo. Its RNA is localised in the Balbiani body of early oocytes, but during early vitellogenesis it is found in the animal hemisphere, eventually dispersing in late oogenesis. A Buc-GFP fusion protein becomes localised to the Balbiani body, and in later oogenesis to vegetal germ plasm, while in embryos the GFP fusion localises in PGCs [15]. Although the protein localisation data depends solely on expression of exogenous Buc-GFP, it is highly likely that the endogenous protein behaves in a similar fashion, although it is not certain when the endogenous protein is actually expressed. While Buc RNA disappears from germ plasm in later oogenesis, it persists in a dispersed state at nearly constant levels through oogenesis and into embryonic development, so it is likely that Buc protein is a constituent of mature zebrafish germ plasm [15].

The *Xenopus* homologue of Bucky ball is Xvelo1. Its RNA is excluded from the mitochondrial cloud in pre-vitellogenic oocytes and in late oogenesis it is localised to the vegetal cortex. As a result this was characterised as late pathway, Vg1-like, localisation [16]. It is expressed as two splice variants. The longer transcript, which we call XveloFL, encodes an 88 kDa protein with no conserved protein motifs which would provide insight into its biological function. There is a shorter splice variant that introduces a frame shift in the C-terminal region (XveloSV), and this RNA has the same temporal expression pattern as the larger transcript [16]. There are Xvelo1/Buc homologues throughout vertebrates, although those in eutherian mammals are more diverged; in humans the RNA lacks a complete open reading frame, suggesting that it has lost its function altogether, or functions as an RNA without translation [15]. Since eutherian mammals lack germ plasm, this fits with a primary role for Buc in germ plasm organisation. An interesting feature of the *Xenopus* Xvelo1 locus is that it overlaps the *polycomb1 (XPc1)* locus, being transcribed in the opposite direction into the extremely long 3′UTR of XPc1 [16].

We have recently reported that fluorescently tagged Hermes protein, like injected fluorescent germ plasm RNAs, is localised into the germ plasm RNPs of vitellogenic oocytes and eggs [4]. Since Hermes is absent from late pathway particles we felt that the identification of Hermes binding partners would shed further light on the special properties of germ plasm. Consequently we have screened for partners of Hermes using the yeast two-hybrid system. This has allowed the identification of several candidate proteins, which include the two Xvelo1 homologues of Bucky ball, and two RNA binding proteins, Rbm24b and Rbm42b. We show that the two protein products of the Xvelo1 gene are naturally present in *Xenopus* germ plasm, in association with Hermes. We also provide evidence that the two Rbm proteins are candidate germ plasm constituents, because they locate sufficiently closely to Hermes in RNP particles to interact in bimolecular fluorescence complementation (BiFC) assays. In addition, depletion of one of them alters germ plasm morphology.

## Results

### Identification of Hermes-binding proteins using the yeast two-hybrid system

The yeast two-hybrid system was used to screen a randomly primed oocyte library [17].,using the Hermes ‘b’ isoform as bait. Table 1 shows positive clones that were isolated in the correct orientation to produce protein and that gave a strong interaction with Hermes, judged by titration with 3-aminotriazole (3-AT) [18]. Of these, three were chosen for further study; Xvelo is a homologue of a zebrafish protein already implicated in germ plasm organisation and Rbm24b and 24b are likely to bind RNA. To validate the initial two-hybrid results, the larger Xvelo insert (containing the first 184 amino acid residues common to both Xvelo splice variants) and those of Rbm24b and Rbm42b were transferred to the LexA DNA binding domain-based bait vector and tested against the Hermes ORF in the pVP16 prey vector. All three clones tested positively on selective media and in β-galactosidase reporter assays.

**Table 1:** Positive clones isolated by the yeast 2-hybrid system, using Hermes b as the bait protein.

Positive Clone	Full length protein (amino acid residues)	Protein region encoded by clone (amino acid residues)	Genbank AccessionNumber
Hermes a	197	1–115	NM_001094266
Hermes b	196	1–111; 7–115; 7–148; ∼1–115*	AF_107889
(4 clones)			
XveloFL/XveloSV (2 clones)	779/269	1–184; 1–156**	AY_280864/AY280865
Rbm24b	224	93–219	NM_001086109
Rbm42b	385	1–174	NM_001097656
ERK5 (2 clones)	925	590–741	AB_213560
Farnesyl transferase, α subunit	379	125–330	NM_001114783
znrd1	122	1–122	XM_002931837***

The positions of sequences encoded by clones are given with respect to their position in the full-length proteins. Hermes “a” and “b” denote the two pseudo-allelic forms of Hermes expressed in *Xenopus laevis*.

* Not fully sequenced.

** This region is common to both splice variants.

*** This is a *Xenopus tropicalis* sequence; no *X. laevis* clone exists in the database.

Interestingly, five of the isolated clones encoded the N-terminal region of Hermes, which agrees with previous findings that wild-type Hermes protein co-precipitates with exogenously introduced tagged Hermes in oocytes [19]. Thus our findings confirm that Hermes is able interact with itself and show that the site for this interaction is located within the N-terminal half of the protein (amino acid residues 1–111).

We also screened a stage 17 neurula library containing much larger inserts, kindly provided by Dr Tim Mohun. While most of the positives did not appear encouraging, one worth noting was Cytokeratin Type I, XK70a. In a previous screen for partners of Xpat, which forms germ plasm particles distinct from those containing Hermes [4], a number of Cytokeratin Type II positives were obtained (Moore and Woodland, unpublished). Cytokeratins are concentrated in the germ plasm and have a role in its anchoring [8], [20].

### Xvelo proteins interact with Hermes in vitro

**Figure 1:**
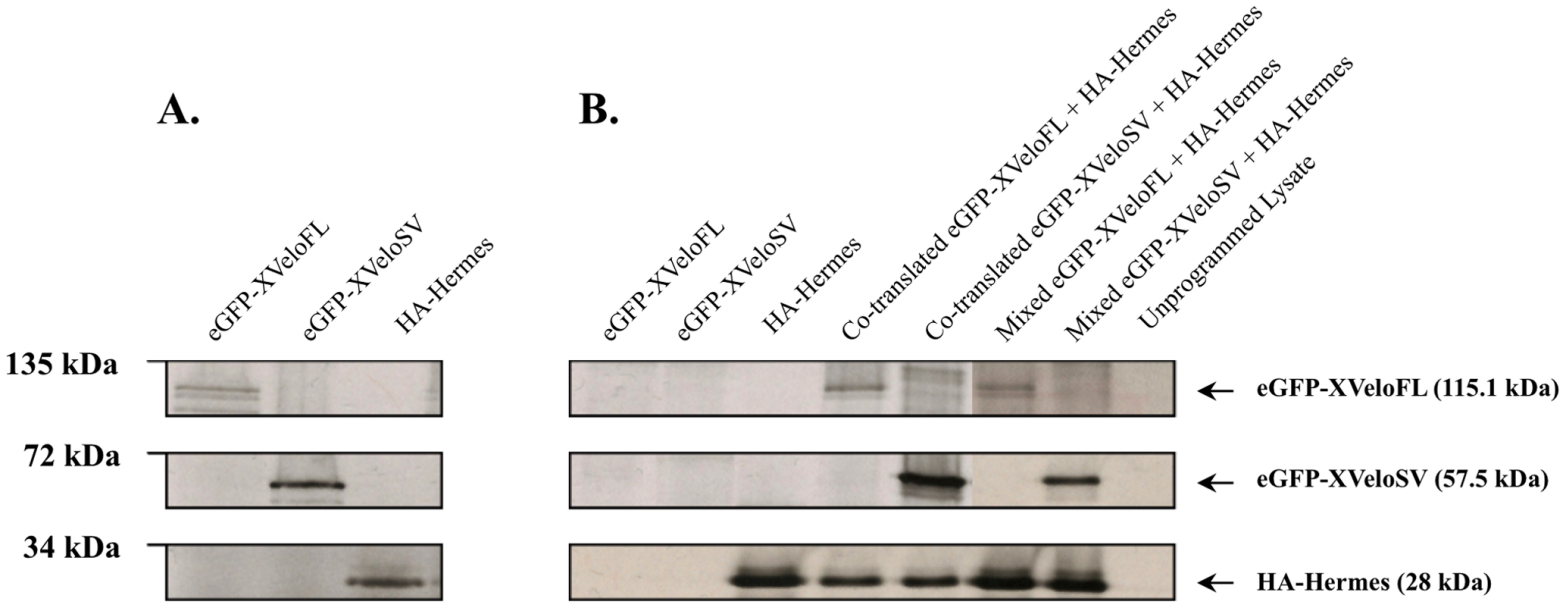
Interaction of in vitro-translated Xvelo1 isoforms with Hermes. (A) eGFP-XveloFL, eGFP-XveloSV and HA-Hermes fusion proteins were prepared in vitro by coupled transcription–translation in the presence of ^35^S-methionine. Translated proteins were resolved by SDS-PAGE and visualized by autoradiography. (B) In pull down experiments translated proteins were added as indicated and immunoprecipitated with a mouse anti-HA monoclonal antibody. Immunoprecipitated proteins were resolved by SDS-PAGE and visualized by autoradiography. The predicted molecular weight for the individual fusion proteins is indicated on the right and marker sizes on the left.

To confirm the interaction of Hermes with the two Xvelo isoforms, fusion proteins encoding eGFP-XveloFL, eGFP-XveloSV (further details see below) and HA-tagged Hermes were prepared by in vitro translation. Both Xvelo proteins were effectively co-immunoprecipitated with Hermes in pull down experiments using anti-HA anti serum (Figure 1). This was true whether the Xvelo and HA-Hermes proteins were co-translated or mixed after translation. Furthermore, the addition of 500 ng of non-translatable *mut-nanos1* RNA [4]did not appear to augment the pull down of the two Xvelo proteins when present in the translation reactions or added later (not shown). Since the constructs encoding each of the fusion proteins lacked their respective wild type untranslated sequences, which are essential for directing their RNAs into RNPs, it seems the proteins themselves interact directly. Further evidence for in vivo oocyte interactions is provided below.

### Do GFP fusions of potential Hermes partners localise to germ plasm?

If the yeast two-hybrid candidates for Hermes partners are biologically significant it is possible that, like Hermes [4] they will localise to germ plasm RNP particles when expressed in oocytes. Although, if they do not, they might still associate with Hermes elsewhere in the cell, or even at different developmental stages. Therefore, we made N-terminal eGFP fusions to the ORFs of XveloFL, XveloSV, Rbm24b and Rbm42b and expressed them in large oocytes by injecting mRNA transcribed in vitro. Germ plasm in the vegetal pole was then examined by confocal microscopy. In a previous study, using the same approach, YFP-Hermes protein was found to localise to a vegetal field of particles, typical of germ plasm. All co-injected Cy5-*nanos1* co-localised in particles with the YFP-Hermes fusion and subsequent staining of the oocytes with Hermes-specific antiserum revealed that it localised with endogenous Hermes. Furthermore, the Hermes RNP particles were interspersed with particles containing the endogenous germ plasm protein Xpat [4].

**Figure 2:**
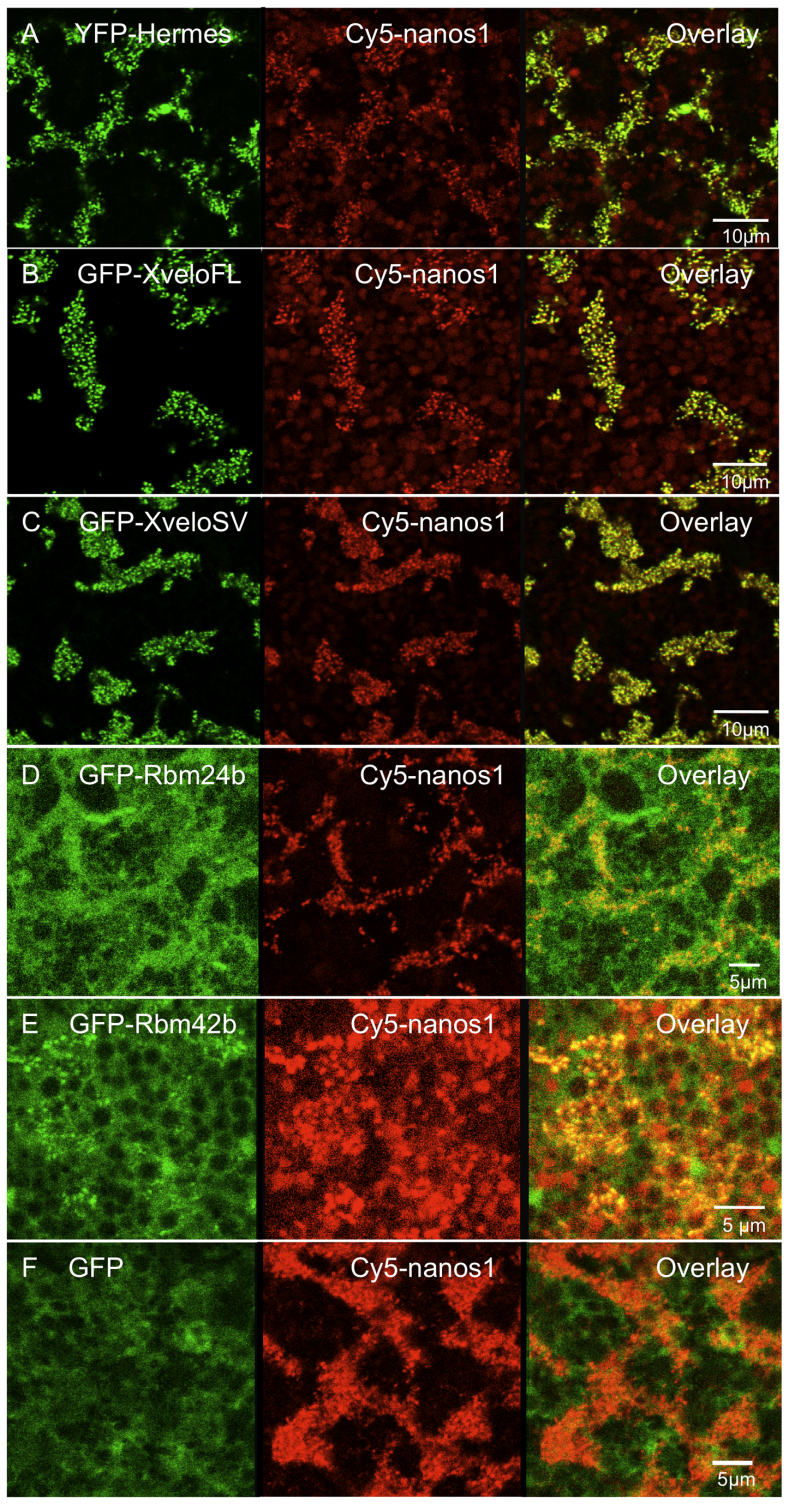
Distribution of GFP-tagged Hermes interacting candidates in the germ plasm region of stage VI oocytes. The vegetal pole of oocytes was injected with Cy5-*nanos1* and mRNAs encoding GFP-tagged proteins as indicated. After culturing in OCM for 48 hours the cortex of live oocytes was examined by confocal microscopy. Overlays are shown on the right. Xvelo proteins localise strongly to germ plasm RNPs, but Rbm42b and particularly Rbm24b localise more diffusely in the general vicinity of the germ plasm. Control RNA encoding GFP protein is shown in F.

RNA encoding GFP-XveloFL was co-injected together with Cy5-*nanos1* mRNA, which serves as a marker of the RNPs in germ plasm [4]. To study the localisation of the proteins, without the added complication of injected RNA localisation, the RNA encoding respective fusion proteins lacked their wild type UTRs and so would not themselves localise. We have previously reported that Cy5-*nanos1* RNA usually entered germ plasm RNPs in full-grown, stage VI oocytes and co-localised with all the Hermes particles [4], as is also seen in Figure 2A. In our previous paper we showed that these Hermes/*nanos1* particles were clearly present in cortical, germ plasm islands, because for example, they were closely interspersed with Xpat protein particles and with aggregated mitochondria [4]. GFP-XveloFL also clearly co-localised with Cy5-*nanos1* in germ plasm RNPs (Figure 2B). Since both YFP-Hermes and GFP-XveloFL were each localised in all of the Cy5-*nanos1* containing particles, YFP-Hermes and GFP-XveloFL must be integral components of the same RNPs. The XveloSV differs from the full-length protein in having different and shorter C-terminal sequences, although these are encoded by a frame shift of the same differentially spliced RNA sequence [16]. We found GFP-XveloSV also co-localised with Cy5-*nanos1* to germ plasm RNPs (Figure 2C). These data are consistent with the idea that both Xvelo proteins naturally form complexes with Hermes and that all three endogenous proteins are present in the same RNP particles.

In the experiments reported here we do not show statistics since each construct consistently gave the localisation pattern illustrated in Figure 2, with the exception of a few negative oocytes in which the injection failed. This applied to many experiments, using different batches of oocytes (Approximately 100 injection experiments using oocytes from different females in the case of YFP-Hermes, tens of experiments for the other proteins). We previously found a minority of oocytes in which germ plasm RNAs were not localised to germ plasm, but this inconsistency was not shown by proteins

We also tested the distribution of GFP-Rbm24b and 42b. The panels in Figure 2D and E respectively show that these fusion proteins localised generally to the germ plasm region, but less specifically than the Xvelo proteins. The two Rbm proteins were also observed to be very strongly and uniformly localised very close to the oocyte surface (not shown). GFP-Rbm24b was present both in germ plasm particles and the intervening space around them. The localisation of the fusion protein was diffuse and expression levels were such that it was difficult to distinguish it from an oocyte injected with RNA encoding GFP alone (Figure 2F) [21]. In addition there were clearly some Rbm24b particles that did not contain Cy5-*nanos1* RNA. Given that Cy5-*nanos1* RNA and Hermes are coincident, Rbm24b might only be a minority component of Hermes-containing RNP particles. Conversely, it could be a more substantial constituent if it is very highly expressed, since fluorescence seen generally around the germ plasm might obscure that present in particles. GFP-Rbm42b was not as strongly expressed as the other proteins from an equivalent amount of injected mRNA, so the levels injected were increased and the resultant expression is shown in Figure 2E. The localised protein was clearly more particulate than Rbm24b and most particles coincided with Cy5-*nanos1* RNA.

Although, we do not have antisera to Rbm24b and 42b, it is possible to estimate the abundance of their mRNAs from EST and microarray expression data on the NCBI and Xenbase [22] sites (http://www.xenbase.org). In the oocyte EST populations Rbm24b (Xl.467) and Rbm42b (Xl.4536) were present at similar frequencies to Hermes, VegT, the Xvelo variants and beta actin, suggesting that their RNAs are quite abundant. This is confirmed by the accurate quantitation of microarray data available on Xenbase, showing that both are abundant maternal RNAs expressed through embryonic development (data derived from [23]).

We also examined the distribution of a GFP-tagged fusion of the farnesyl transferase α subunit identified during the two-hybrid screen (Table. 1). While the protein was present in the germ plasm region, it was difficult to distinguish it from oocytes injected with RNA encoding GFP alone (not shown) and therefore it was not investigated further.

### Confirmation of proposed protein interactions by bimolecular fluorescence complementation (BiFC)

Co-localisation indicates the potential for interaction, but it does not prove that it occurs. We have attempted to add to the evidence of interaction in yeast by using BiFC. In this technique two non-fluorescent moieties of YFP, or a derivative, are tethered to potential interacting partners which, when brought into close apposition, interact to form a fluorescent protein. There is a background rate of spontaneous fluorophore self-assembly associated with the technique, but we used mutants constructed by Saka et al. [24] in which this is minimised. These mutants were based on the YFP variant VENUS, to give increased fluorescence. While BiFC detects molecular interactions, the VENUS association is relatively irreversible [25], so there may be over-representation of interacting forms of the proteins. Nevertheless the complexes detected should certainly normally exist within the oocyte, provided the proteins are naturally expressed there. Each candidate protein was N-terminally linked to both the N- and C-terminal moieties of VENUS (VN and VC respectively) and each combination of potential partners was tested with both moiety pairs, with the exception of Rbm24b/24b, 42b/42b and 24b/42b.

**Figure 3:**
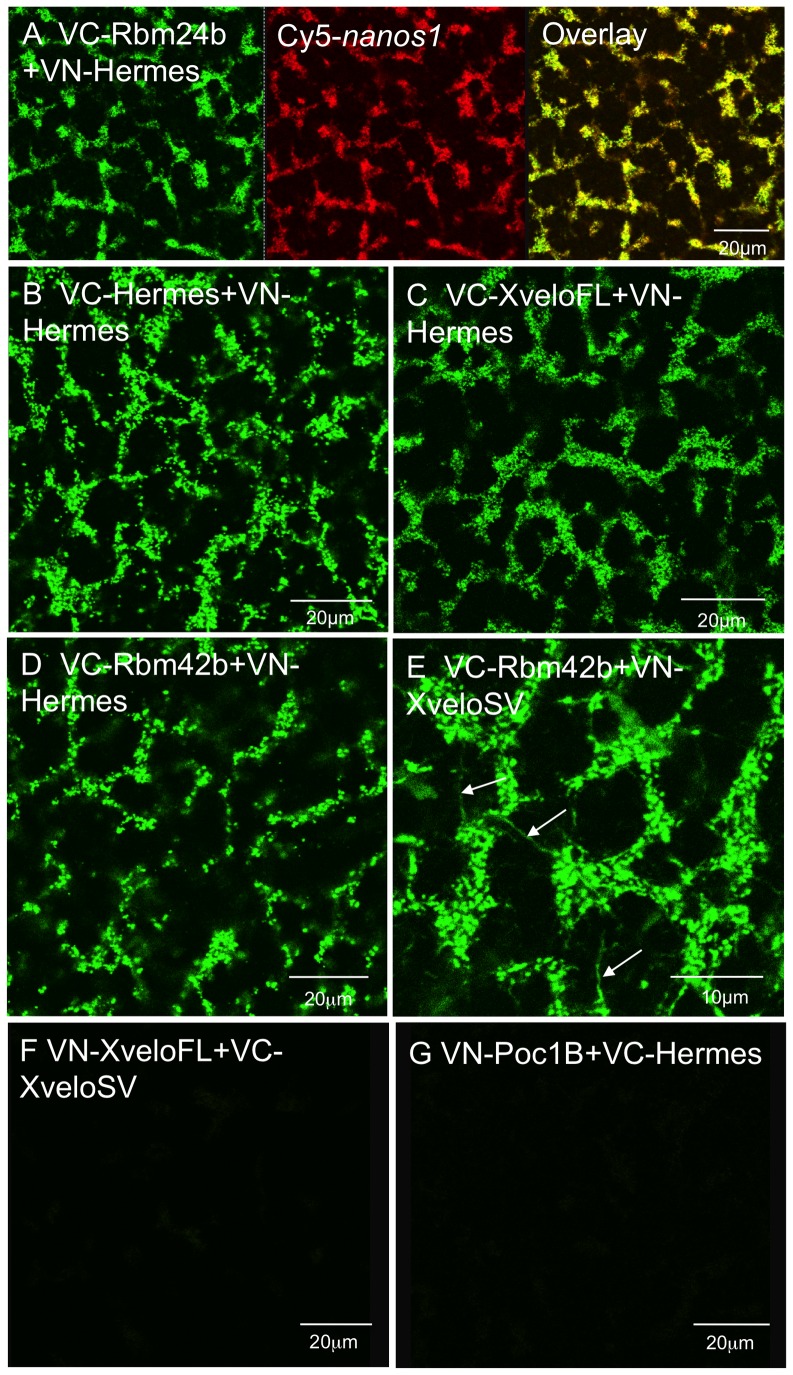
Demonstration of in vivo protein-protein interactions of Hermes and its partners by BiFC analysis. Candidate interacting proteins were expressed in stage VI oocytes by injecting mRNAs expressing the C-terminal (VC) and N-terminal (VN) fragments of Venus fused to the N-terminus of the two candidate proteins. Oocytes were then cultured for 48 hours in OCM, after which the vegetal cortex of live oocytes was examined by confocal microscopy. Constant gain settings were used in all instances, and with these settings non-interacting controls, such as Poc1B/Hermes (Panel G) or other negative combinations (panel F) gave no significant signal. In all cases Cy5-*nanos1* was also co-injected and was coincident with the green fluorescence, but this is shown only in A.

Since there is always a small degree of reassociation of the VENUS moieties, regardless of the specific interactions of candidate partners, all comparisons were made using constant gain settings during confocal microscopy. In Figure 3A-E we see the strong signals produced by Hermes interacting with itself, as well as with some of the other proteins studied here. It is noteworthy, that in all instances where interactions were identified in the germ plasm, co-injected Cy5*-nanos1* RNA was always coincident, e.g. Figure 3A. To provide a null signal we chose *Xenopus* Poc1B (previously called xPix1). This is a germ plasm component which co-localises with Xpat protein in particles that do not contain Hermes [4], [10]. At the constant gain settings shown in Figure 3 there was almost no detectable signal from oocytes co-injected with RNA encoding VC-Hermes and VN-Poc1B, or the reverse combination (Figure 3G). The same was true of a test for Poc1B homodimerization using VN and VC derivatives of Poc1B (not shown). The totality of combinations tested, and the resultant homo/heteromeric interactions identified are shown in Table 2. Hermes clearly interacts with itself, as originally reported by Gerber et al. [19]. All of the candidates analyzed here interact with Hermes in germ plasm, validating our initial yeast 2-hybrid data (Table 1). In addition the larger Xvelo protein (XveloFL) interacts with itself and strongly with Rbm42b, but more weakly with Rbm24b. On the other hand it did not interact with XveloSV, nor did this interact with itself.

**Table 2:** Protein-Protein interactions of Hermes binding partners in Bimolecular fluorescence complementation (BiFC) experiments.

	VC-Hermes	VC-XveloFL	VC-XveloSV	VC-Rbm24b	VC-Rbm42b	VC-POC1B
**VN-Hermes**	Very Strong, GP, PN	Strong, GP, PN	Very Strong, GP, PN	Strong, GP, N, CP	Very Strong, GP, N, CP	None*
**VN-XveloFL**		Strong, GP, PN, CP	None	Strong, GP, PN, CP	Very Strong, GP, PN, CP	ND
**VN-XveloSV**			None	Strong, GP, PN	Very Strong, GP, N, PN, CP	ND
**VN-Rbm24b**				Strong, GP, PN, CP	Strong, GP, CP	ND
**VN-Rbm42b**					ND	ND
**VN-POC1B**						None

Interactions were visualised in stage VI oocytes by co-injecting RNA encoding the fusion proteins indicated. VN and VC denote the N-terminal and C-terminal fragments of Venus respectively, fused to the N-terminus of the proteins tested. The strength and sites of sub-cellular interactions are shown. (GP, germ plasm; N, Nuclear; PN, perinuclear; CP, cytoplasmic particles; ND, not determined). *This was the defined negative background signal.

How does one know that negative results are not the result of low expression? An example of the justification is illustrated by the VN-XveloFL/VC-XVeloSV combination. While this results in no detectable interaction (Figure 3F), the same levels of RNA encoding each of these proteins gave strongly positive results when co-injected with VC/VN Rbm24b/42b RNAs, showing that both proteins are efficiently expressed.

One combination is worthy of further comment. Interestingly Figure 3E shows that the VC-Rbm42b interaction with VN-XveloSV labelled fibres extending from and between germ plasm islands, presumably protein associated with the cytoskeleton (white arrows. Figure 3E). It is possible that these represent transport intermediates in the germ plasm region.

### Distribution of Hermes, its partners and their complexes in the oocyte interior

**Figure 4:**
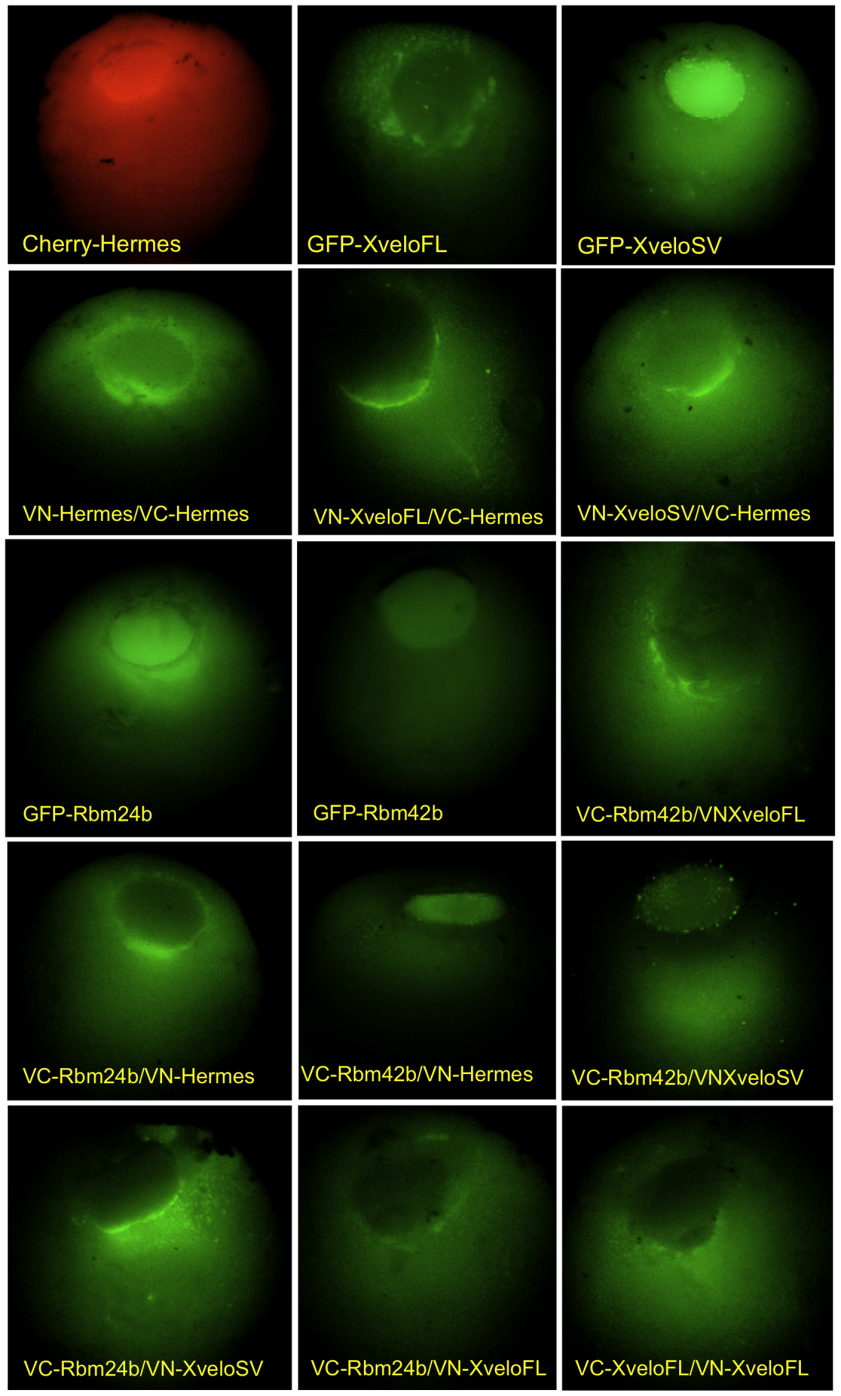
Sub-cellular localisation of GFP fusion proteins and of the sites of where they interact. Stage VI oocytes were injected with mRNA encoding the constructs indicated, and cultured in OCM for 48/1% formaldehyde at -20°C overnight, rehydrated and sectioned in an animal/vegetal plane by hand. Low power fluorescent stereo microscope images are shown.

**Figure 5:**
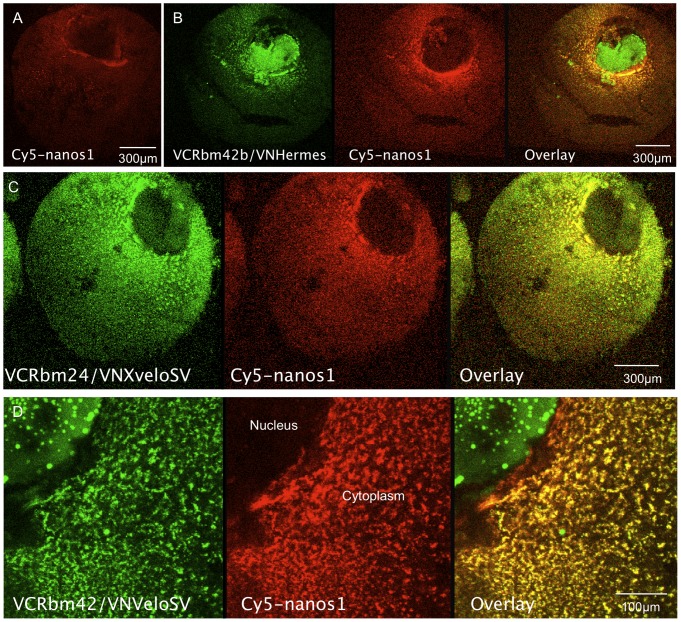
Confocal analysis of BiFC constructs co-injected with labelled nanos1 mRNA. Sectioned stage VI oocytes, prepared as in Figure 4, were imaged in the confocal microscope. Sections made by hand with a scalpel are quite irregular, so Z-stacks are shown. Panel (A) shows an oocyte injected with Cy5-*nanos1* RNA alone.

So far we have focused on the localisation of proteins to germ plasm in the oocyte cortex. Since confocal analysis of whole full-grown oocytes only allows visualisation within about 20 µm of the oocyte's surface, we manually sectioned fixed oocytes and analyzed them with a fluorescence stereo microscope to establish the internal distribution of fluorescent proteins. The highly reproducible results are shown in Figure 4 (when visualized in this way the very thin germ plasm component is usually not seen). YFP or mCherry-tagged Hermes was intensely localised within the nucleus and in a perinuclear region, mostly on the vegetal side of the nucleus, an area of concentrated microtubules [26]. There were also fields of particles throughout the cytoplasm spreading towards the oocyte periphery. The confocal images in Figure 5 illustrate the fact that all the expressed proteins and Cy5-*nanos1* RNA were present as particles, comparable to those in germ plasm.

GFP fusions of XveloSV, Rbm24b and Rbm42b (Figure 4) all showed nuclear concentration, and Rbm24b was also abundant in the perinuclear region. On the other hand GFP-XveloFL, the larger Xvelo protein, was concentrated in the perinuclear region, rather than the nucleus. Within the nucleus no complexes were present as small particles, although XveloSV plus Rbm42b did form larger spherical structures. These may possibly be over-expression artefacts, but alternatively they could be Cajal or coiled bodies [27], [28]. However, this is purely speculative and needs further work.

The BiFC expression constructs revealed interesting differences between the individually expressed proteins and their complexes (Table 2). As mentioned already, Hermes was concentrated in the nucleus. However there was little if any Hermes/Hermes BiFC signal within the nucleus; rather the self interaction occurred in the cytoplasm, particularly around the nucleus, as well as in germ plasm (see above). This suggests that Hermes may have an alternative RRM-containing partner within the nucleus (possibly Rbm42b, see below). For XveloFL the self interaction was the same as the distribution of the GFP-protein, i.e. it was extra-nuclear. XveloSV did not give an interaction signal with itself, nor with XveloFL (not shown), as was also true in germ plasm (see above). This suggests that Xvelo self interactions rely on the C-terminal regions present in XveloFL. Although GFP-XveloSV and mCherry- or GFP-Hermes were intensely nuclear, they interacted only in the cytoplasm. A possible reason for this is that XveloSV only interacts with Hermes homomultimers. Exactly the same applies to Rbm24b, but Rbm42b interacted with Hermes in the nucleus, but not at high levels in its periphery. As already mentioned, Rbm42b and Hermes also interacted in germ plasm, as did Hermes and Rbm24b. It is interesting that, while XveloSV and Rbm24b were both concentrated in the nucleus, they interacted only in the cytoplasm. If Hermes needs to multimerise with another RRM-containing protein, it may be that Rbm42b fulfils this role in the nucleus, whereas all the RRM proteins (i.e. Hermes, Rbm24b and Rbm42b) interact in the cytoplasm.

We co-injected the BiFC constructs with Cy5-*nanos1* to establish how the BiFC particles related to those containing *nanos1*. All of the interacting proteins co-localised with *nanos1* RNA in cytoplasmic particles; Figure 5 shows several examples. RNA injected on its own (Figure 5A) had the same distribution as when co-expressed with proteins, so the over-expressed proteins did not influence RNA distribution. As mentioned earlier, the BiFC-interacting particles also all co-localised with the RNA in the germ plasm (Figure 3A).

The possible significance of these diverse localisation patterns is discussed later.

### Antiserum staining shows that endogenous XveloFL and SV are naturally present in germ plasm RNPs

Expressing GFP-labelled proteins demonstrates their potential for localisation, but does not prove that endogenous proteins are distributed in the same way, since they might not be expressed at all. However, if they are expressed, it is very likely that the GFP fusions are informative. It is already known from antiserum staining that endogenous Hermes is expressed in oocytes and is localised to germ plasm [11] and, the expression characteristics of a YFP-Hermes fusion protein are identical to the wild type protein in the cortex [4]. Thus our data on YFP-Hermes should also show the actual distribution of this protein in the oocyte interior. To establish if Xvelo is expressed in oocytes we raised goat antibodies against unique regions of both XveloFL and SV. Antibodies were then affinity purified using fragments of the full-length proteins containing the synthetic peptide sequences used for immunisation, expressed in *E. coli*.

**Figure 6:**
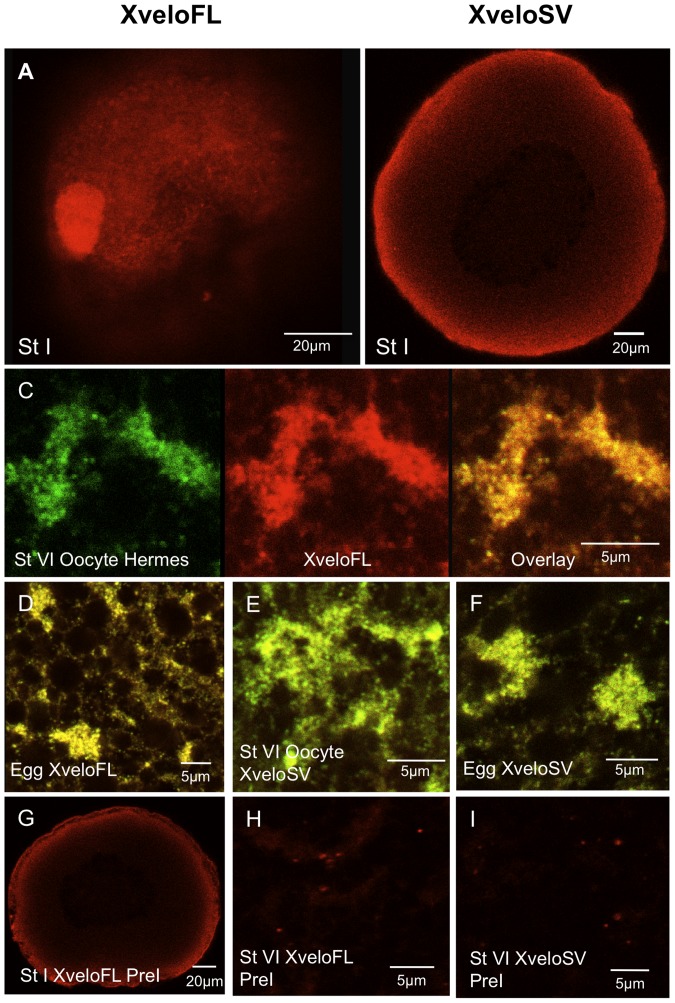
Expression of endogenous Xvelo isoforms during oogenesis. Oocytes, isolated with collagenase treatment to remove follicles, were fixed and stained with affinity-purified antibodies prior to confocal microscopy. (A–B) Previtellogenic oocytes stained for XveloFL and SV respectively. (C–F) The germ plasm region of stage VI oocytes and fertilized eggs. In (C) Hermes and XveloFL were stained and we show coincidence in an overlay in the right hand panel. In (D–F) only the overlays of Hermes (green) and Xvelo proteins (red) are shown. C) and (E) show stage VI oocytes and (D) and (F) show fertilized eggs. Alexa Fluor 488 and Dylight 594 conjugates were used as secondary antibodies for Hermes and Xvelo protein-stained oocytes respectively. Pre-immune serum for both XveloFL and SV is shown in panels G–H. The data was identical when secondary antibodies alone were used (not shown).

These antibodies proved ineffective in western blots of endogenous proteins, although they did detect over-expressed fusion proteins. Part or most of the problem may be that it is very difficult to solubilise Xvelo proteins and we were unable to purify germ plasm particles. However, the antibodies did work well in immunofluorescence staining of oocytes. We find that both isoforms of the endogenously expressed Xvelo protein are localised in the germ plasm RNPs of full-grown oocytes and fertilised eggs, where they are co-incident with Hermes (Figure 6). However, only XveloFL is localised to the Balbiani body of pre-vitellogenic oocytes. As we previously reported [4], the size of germ plasm islands radically decreases during oocyte maturation and the progression to fertilisation. At high resolution the morphology of the Xvelo-labelled islands in fertilized eggs (Figure 6D,F) is like that seen with other germ plasm markers, e.g. Xpat and Hermes [4], [9]. The appearance and behaviour of exogenously expressed GFP-Xvelo fusions during in vitro oocyte maturation was found to be identical (not shown).

The specificity of the XveloSV antibody was confirmed by depleting XveloSV RNA and using the antibody to see if the XveloSV protein signal was reduced. The reduction was very marked, and is dealt with in a later section. This approach was not possible for XveloFL, because the protein is very stable, both in its native and GFP tagged forms (see below). It is no surprise that such proteins are so stable, since they are integral components of storage complexes formed as the oocyte matures over a period of several months. Also this apparent stability is not unique to XveloFL and may be a widespread property of Balbiani body-associated proteins, since the same problem was previously encountered with Xpat and Poc1B (Pix1) proteins [9], [10].

When oocytes were injected with GFP-XveloFL or GFP-XveloSV and stained with the respective antibodies, the resultant GFP and immuno-fluorescent signals were found to be coincident (not shown). This indicates that in the cortex the antibodies stain only germ plasm particles containing the Xvelo variants.

In conclusion it is clear that Hermes, XveloFL and XveloSV are constituents of the same germ plasm RNPs and that these are identical to all particles containing injected *nanos-1* mRNA.

### The N-termini contain the peptide sequence(s) essential to localise Xvelo proteins to germ plasm particles

**Figure 7:**
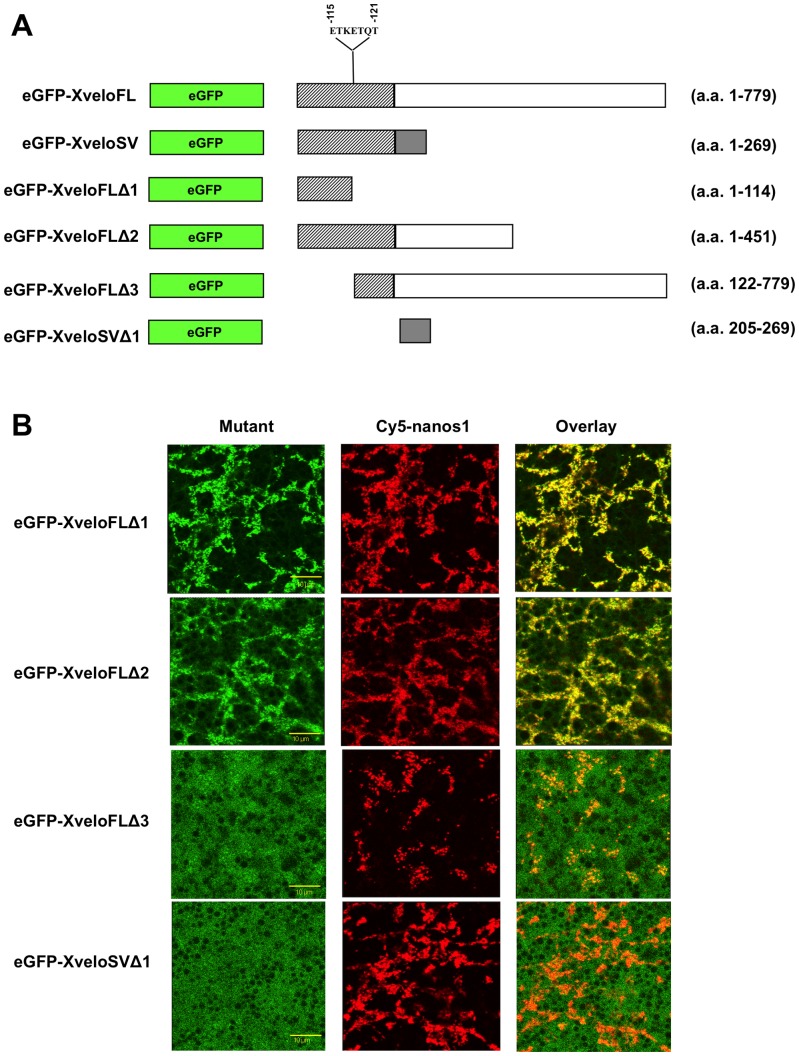
Localisation of Xvelo mutant proteins. (A) Schematic diagram of GFP-tagged Xvelo mutant constructs. Hatched boxes represent amino acid residues present in both XveloFL and XveloSV proteins. Clear and grey boxes represent amino acid residues specific to XveloFL and XveloSV proteins respectively. The position of a putative Dynein light chain 8 (DLC-8) interacting motif, identified using the ELM software (elm.eu.org) database is also shown. (B) Stage VI oocytes were co-injected with Cy5-*nanos1* RNA and RNA encoding the indicated mutant Xvelo protein. Oocytes were cultured for 48 hours in OCM and localisation was then assessed by confocal microscopy.

To establish the location of Xvelo sequences needed for germ plasm localisation we expressed a series of deletion mutants in oocytes (Figure 7A; Table 3). It is clear that sequences in the N-terminal region common to both Xvelo isoforms are sufficient to localise the proteins into germ plasm particles. The shortest Xvelo clone identified by the yeast two-hybrid screen encoded amino acid residues 1–156. Thus this contains the region that interacts with Hermes and it is possible that anchoring to Hermes brings about the localisation of Xvelo, or indeed the converse. The first 114 amino acids contained within the mutant eGFP-XveloFLΔ1 alone promoted strong localisation (Figure 7B).

**Table 3:** Summary of experiments testing the localisation of Xvelo proteins and mutants.

	Localisation exclusively to germ plasm particles	No localisation
**eGFP-XveloFL (a.a. 1–779)** (N = 4)	25/25	0/25
**eGFP-XveloSV (a.a.1–269)** (N = 3)	19/20	1/20
**eGFP-XveloFLΔ1 (a.a. –114)** (N = 2)	16/16	0/16
**eGFP-XveloFLΔ2 (a.a. 1–451)** (N = 2)	10/10	0/10
**eGFP-XveloFLΔ3** * **(a.a. 122–779)** (N = 4)	23/28*	5/28
**eGFP-XveloSVΔ1 (a.a. 205–269)** (N = 2)	0/14	14/14

* eGFP-XveloFLΔ3 localisation was always very weak, and only discernable at very high gain settings during confocal microscopy. If localisation ability was strictly based on the criteria used for the other full length proteins or mutants (ie constant lower gain) they would probably all be scored as negative.

N is the number of experiments using different batches of oocytes The number of oocytes displaying germ plasm localisation are scored with respect to the total number injected. For eGFP-XveloFL and eGFP-XveloSV the numbers shown are exclusively for experiments in which the mutants were also tested. Similar data were obtained for these proteins in a much larger number of experiments.

**Table 4:** Quantification of Xvelo isoforms in oocytes injected with antisense oligonucleotides.

	XveloSV antibody		XveloFL antibody	
Antisense oligo	Fluorescence in individual oocytes (mean and standard deviation)	Inhibition/t-test p value (sample versus control)	Fluorescence in individual oocytes (mean and standard deviation)	Inhibition/t-test p value (sample versus control)
Control	36,97,110,60. Mean 76±34.	NA	119,73,122,86. Mean 100±24.	NA
ASXveloSV	28,49,28,47,10,24. Mean 31±15.	59% p = 0.0098	24,46,115,88,33. Mean 61±39.	39% p = 0.063
ASXveloFL	78,83,110,107,99. Mean 95±14.	−25% (i.e. stimulation) p = 0.137	55,39,72,98. Mean 66±25.	34% p = 0.050
ASXveloFL/SV	26,33,48,35. Mean 36±10	53% p = 0.031	45,137,55,68,55. Mean 72±37.	28% p = 0.119

**Figure 8:**
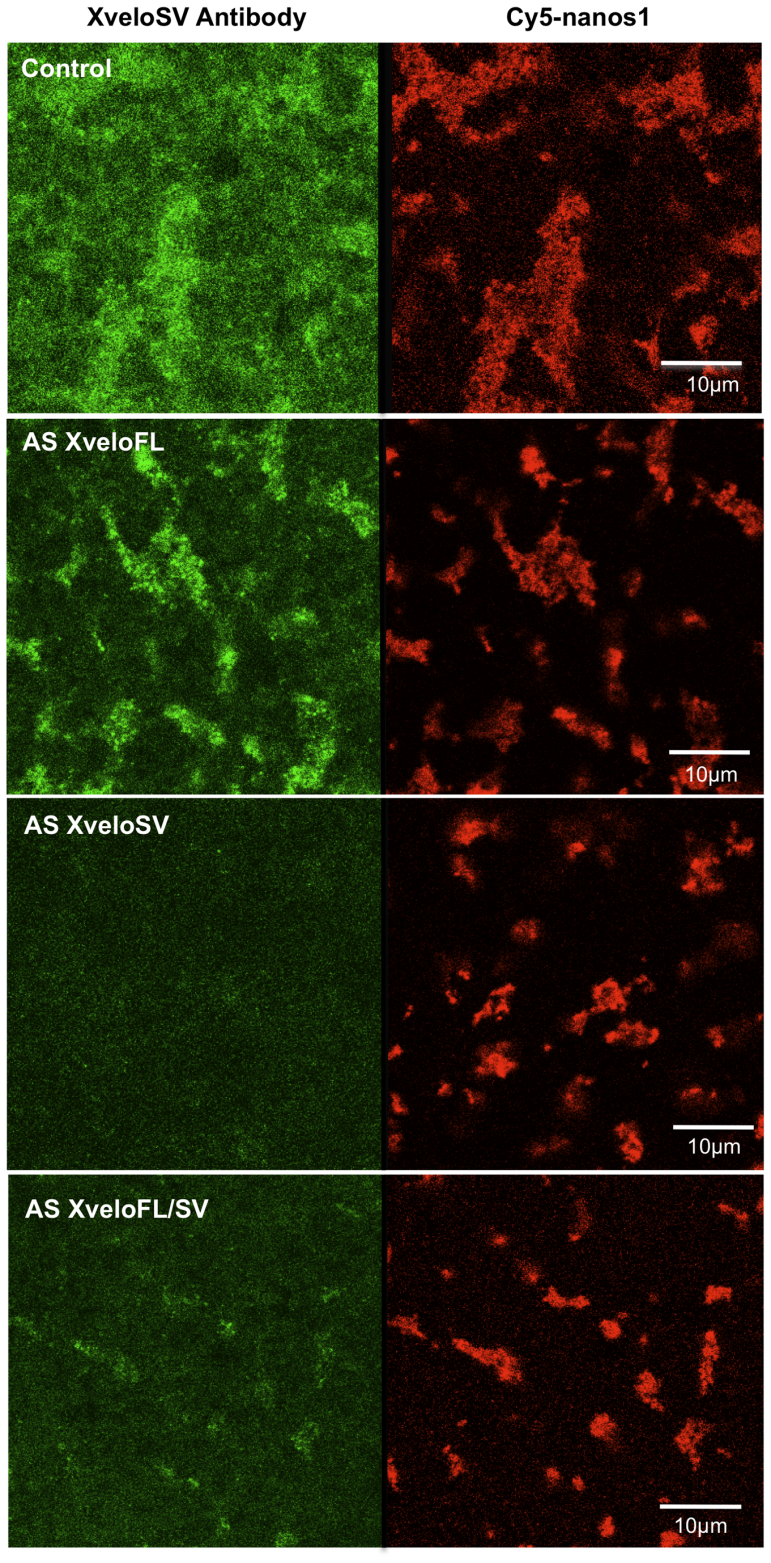
Depletion of endogenously expressed XveloSV protein by anti sense (AS) DNA oligos. Stage VI oocytes were co-injected with Cy5-*nanos1* RNA and 11 ng of the indicated AS-oligo. Following culture in OCM for 48 hours, oocytes were fixed, and stained with purified anti XveloSV antibodies using an Alexa Fluor 488 conjugated secondary antibody. The germ plasm region was then examined by confocal microscopy. The co-injected AS-oligo is indicated in each panel.

For each analyzed oocyte (in the same experiment yielding the data in Figure 8) ImageJ [32] was used to calculate mean RGB brightness values over areas of four germ plasm islands; background was measured over four areas; these were averaged and the background subtracted from the island average. This is shown in the second and fourth columns. Only the XeloSV fluorescence of the ASXveloSV and ASXveloFL-SV oligo-injected oocytes is significantly different from the controls at greater than the 95% level, as judged by the one tailed Student t-test.

As mentioned earlier, previous studies on *Xenopus* Xvelo1 and its zebrafish homologue failed to identify any conserved protein domains, and hence any insights into its biological function. Interestingly, using the ELM software database (elm.eu.org) we were able to identify a putative DLC-8 interaction motif (ETKETQT, residues 115 to 121 inclusive) located in the N-terminus of the protein. Another germ plasm protein, Germes, has been shown to interact with dynein light chain (DLC-8a and b) [21], possibly indicating a common requirement for binding to microtubule motors to facilitate germ plasm localisation. However, in stage VI oocytes this DLC-8 motif does not appear to be required for localisation to germ plasm, since the mutant eGFP-XveloFLΔ1 (which lacks this motif) localised very efficiently (Figure 7B). This does not necessarily mean that the DLC-8 motif is unimportant. Germ plasm particles are large and must contain many molecules. Thus a mutant protein might localise into the germ plasm particles because of interactions with its endogenous non-mutant counterpart, and the endogenous protein itself could continue to perform roles in anchoring proteins into RNP particles or in positioning these particles.

What is clear is the requirement of the first 121 amino acid residues of Xvelo for its efficient localisation to the germ plasm in stage VI oocytes. This is illustrated by the fact that the mutants eGFP-XveloFLΔ3 and eGFP-XveloSVΔ1, which lack this sequence, fail to localise efficiently, although the former localises to some degree and the latter still retains its ability to localise to the nucleus (data not shown).

### Depletion of XveloSV with antisense DNA oligonucleotides reveals a role in the maintenance of germ plasm structure

To prove that the identified interacting proteins have an important function in germ plasm structure and positioning, we used antisense DNA oligos to destroy endogenous RNAs [29]. Of course this will only deplete proteins if they are unstable. For *XveloFL* and *SV* we made isoform-specific oligos. Since *XveloSV* contains sequences present in *XveloFL* except for one internal exon we designed a specific *XveloSV* oligo spanning the splice junction (AS XveloSV). Complimentary oligonucleotides were also designed to the unique sequence in *XveloFL* (AS XveloFL) and to a region common to the two RNAs (AS XveloFL/SV). RT-PCR showed that these oligos were effective in depleting their cognate messages (not shown).

We used the Xvelo isoform-specific antisera described in the last section to assess protein depletion of Xvelo variants by the antisense oligos, using confocal microscopy (Figure 8). XveloFL protein localised to germ plasm showed no detectable diminution with either the specific oligo (AS-XveloFL) or that recognising the two Xvelo variants (AS XveloFL/SV) (not shown). However, while XveloSV protein was unaffected by the oligo specific to XveloFL, its abundance in germ plasm islands was reduced following injection of the specific AS XveloSV oligo, and somewhat less by the oligo recognising both types of transcript (AS XveloFL/SV; Figure 8). The use of two entirely different oligos is important in eliminating off target effects. The amounts injected are similar to those used by Janet Heasman, Chris Wylie and colleagues in the host transfer technique [30]. These permit subsequent normal development of embryos, so long as an essential RNA is not depleted. We quantified the reductions achieved by the oligos by analysing confocal images like those in Figure 8, using Image J software [31] (Table 4). This is not very accurate, most likely because the staining of individual oocytes with antisera varies in efficiency. Only the reduction of XveloSV expression by AS XveloSV and AS XVeloFL/SV are statistically significant. It seems that the amount of XveloSV protein in germ plasm granules is, on average, reduced by about half.

Because we did not have antibodies to the Rbm proteins, we designed a strategy to test depletion based on expression of their GFP fusions. We injected mRNAs encoding these proteins and cultured the oocytes for 24 h prior to injecting antisense oligos. This was also applied to GFP fusions of the two Xvelo proteins described earlier (Figure 2). The results obtained with tagged XveloFL and SV proteins were similar to those seen with antibody detection of endogenously expressed proteins following antisense oligo injection. That is, GFP tagged XveloSV protein was depleted, but XveloFL was not. There was no obvious decrease in Rbm24b and Rbm42b GFP fluorescence. We have not shown these negative data.

**Figure 9:**
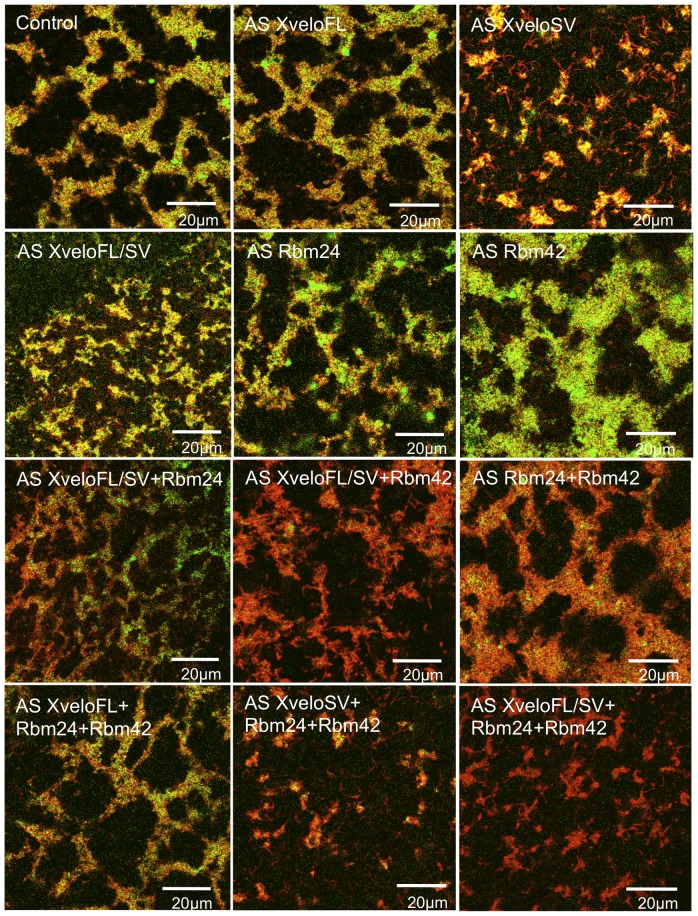
Effect of RNA depletion on the size of germ plasm islands and the localisation of YFP-Hermes. Stage VI oocytes were injected with RNA encoding YFP-Hermes plus 11 ng of each antisense oligo. After culturing for 48 hours in OCM, mitochondria were stained with TMRE and the germ plasm region examined by confocal microscopy. Overlays of YFP-Hermes (green) and mitochondria (red) are shown, and the AS oligos used indicated on each panel.

It is clearly apparent that oligos targeting *XveloSV* mRNA reduced the size of the germ plasm islands (Figure 8). Furthermore, we found that the consequence of XveloSV depletion applied to mitochondria in germ plasm islands, as well as to *nanos1* RNA localisation (Figure 9). Specifically, mitochondrial aggregates were seen to be significantly smaller, with numerous mitochondria no longer tightly localised within them. Thus XeloSV appears to have a general role in compacting germ plasm islands.

As expected from its ineffectiveness at protein depletion, AS XveloFL had little phenotypic effect, consistent with the AS XveloSV phenotype being a specific consequence of XveloSV RNA depletion, rather than simply oligo injection (Figure 8). Furthermore the common AS XveloFL/SV oligo produced a phenotype similar to that seen with AS XveloSV, although it was slightly attenuated, consistent with its somewhat less efficient depletion of protein. The fact that two independent oligos have the same specific effect suggests that an off target effect is not involved. A general effect of toxicity is unlikely since combinations of oligos at three times the concentration of AS XveloSV alone, do not affect germ plasm island size, unless they contain AS XveloSV (see below).

AS Rbm24 and AS Rbm42 oligos had no effect on germ plasm morphology, as one might expect from their inability to deplete their respective GFP fusion proteins significantly. However, it is possible that there are small depletions mediated by these oligos, which when combined could synergise to produce a phenotype. Figure 9 shows the effect of injected oligo combinations on germ plasm structure. It is notable that when AS XveloSV was combined with AS Rbm24, and particularly AS Rbm42, the localisation of the co-expressed germ plasm marker YFP-Hermes, was severely impaired. The two Rbm AS oligos also had this effect when combined, although the islands were not reduced in size without depletion of XveloSV. A small reduction in the total amount of these proteins might have a significant effect on their combined spare Hermes-binding capacity. A control for the specificity of the observed germ plasm changes was provided by AS XveloFL, which when combined with AS Rbm24 and 42 had no effect on island size.

**Figure 10:**
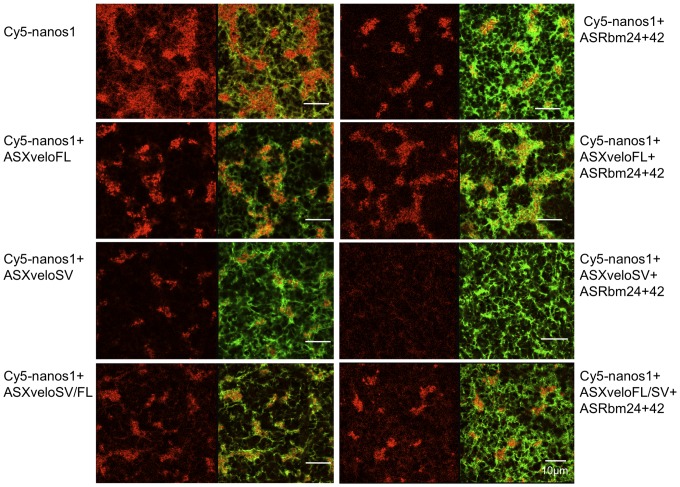
RNA depletion by antisense oligos does not affect the distribution of microtubules in the germ plasm region. Stage VI oocytes were co-injected with Cy5-*nanos1* RNA and 11 ng of each of the AS oligos indicated. Oocytes were then cultured in OCM for 48 hours, fixed and stained with a rabbit anti-α-Tubulin polyclonal antibody #2144 (Cell Signaling Technology) and a goat anti rabbit Alexa Fluor 488 conjugated secondary antibody, prior to confocal microscopy. Panels show Cy5-nanos1 (red) left, and an overlay of this with microtubules (green) right. Scale Bar, 10 µm.

These combinations lead to the tentative conclusion that Rbm24b and 42b may have a role in the recruitment of Hermes to germ plasm particles. More importantly, XveloSV was essential for the aggregation of particles into large germ plasm islands, and more surprisingly for accumulating mitochondria there, even though only about 50% of the protein was depleted from germ plasm particles (Table 4). If completely removed the phenotype could well be more extreme. Bearing in mind that XveloFL was not significantly depleted in these experiments, it is conceivable that it and XveloSV play largely redundant roles, and that the two splice variants together are essential for germ plasm island formation and maintenance. It is possible that depletion of XveloSV plus Rbm24b and 42b also reduce Cy5-*nanos1* incorporation into germ plasm (Figure 10), but more work is needed to confirm this.

The structure of germ plasm is affected by inhibiting microtubule polymerisation since colcemid or nocodazole treatment leads to a running together of germ plasm islands [4]. Therefore, it is conceivable that depletion of RNA and or protein by the AS XveloSV oligo, affects microtubule organization. However, staining of microtubules after AS oligo injection revealed no significant effect on the distribution of microtubules (Figure 10). Since mitochondria were also less aggregated following injection of AS XveloSV, it seems that the overall structure giving coherence to germ plasm islands was disrupted in a micro-tubule-independent fashion.

## Discussion

The single RRM-containing protein Hermes is present in the RNP particles of mature *Xenopus* oocyte germ plasm, as well as in their precursors in the earlier mitochondrial cloud [4], [11]. Although it is present elsewhere in the oocyte [11], including the nucleus, it is absent from late pathway particles of the vegetal cortex [4]. Thus we argued that identifying Hermes binding partners would help us to understand the special nature of germ plasm RNA storage units. Here we report a number of candidate proteins that interact with Hermes and provide evidence, in some cases, that these interactions actually occur in germ plasm, or in other instances that they could do so if the proteins are naturally expressed in oocytes. The fact that these interactions occur in yeast, in the presence only of fragments of the encoding mRNAs, shows that the protein interactions detected are independent of specific interactions with oocyte RNAs.

### Xvelo1 proteins are found in germ plasm RNPs and may play a structural role in germ plasm islands

Xvelo1 was originally identified as an RNA localised to the vegetal pole of the full-grown oocyte [16]. As explained in the Introduction, genetic evidence shows that its zebrafish homologue, Bucky ball (Buc), is essential for oocyte polarity, as well as Balbiani body and germ plasm formation, although it is unclear how it does so. Moreover, when over-expressed in the embryo, additional ectopic PGCs were formed, suggesting an indispensable and directive role for Bucky ball in germ plasm organisation in the zebrafish [15]. While Bucky ball is needed to make the Balbiani body in this species, its role in the formation of the homologous structure found in man is enigmatic [32], since the human gene homologous to *bucky ball/Xvelo1* is interrupted by stop codons [15]. In this case it is possible that other redundant components may be able to substitute for it in forming a Balbiani body on an evolutionary timescale. Of course humans do not form germ plasm and it is not at all clear what the function of mammalian Balbiani bodies might be. However, the loss of Bucky ball protein coding ability in humans fits with a role in germ plasm RNP particle formation, since there is no evidence for RNA localisation in mammalian eggs. In zebrafish *bucky ball* RNA is present throughout oogenesis, but the expression characteristics of its protein are uncertain. Although, a Buc-GFP fusion enters the Balbiani body of early oocytes and the germ plasm of embryos, the endogenous protein has not been studied. However, the fact that the *bucky ball* mutation was only rescued by a translatable message suggests that Bucky ball protein is an essential player in germ plasm formation.

*Xenopus* Xvelo1 exists as two splice variants, which we show interact with Hermes through their common N-terminal region. Both GFP-Xvelo fusions enter germ plasm in large oocytes and both (as well as YFP-Hermes) enter the Balbiani bodies of previtellogenic oocytes (data not shown). Staining of oocytes with Xvelo isoform-specific antisera demonstrates that both proteins are present in the RNP particles of germ plasm in large oocytes and fertilized eggs, where they co-localise with Cy5-*nanos1* RNA in a distribution identical to their respective GFP fusions. However, only the larger variant, XveloFL, appeared to be naturally present in the earlier mitochondrial cloud.

BiFC experiments supported the conclusion that Hermes interacts with XveloFL and SV in germ plasm RNPs and that this is not simply because they are packed into these structures. Positive BiFC interactions between two candidate proteins may occur when they are less close than is required for FRET, i.e. up to 10 nm [33], so one might wonder if simply being packed into the same RNP particle is sufficient to give a positive signal. The negative results obtained using BiFC constructs for XveloSV and XveloFL reveal that this is not the case (Figure 3F, Table 2). Although, GFP fusion proteins for XveloFL and SV must localise into the same RNPs (i.e. both are found in all of the particles that accumulate labelled Cy5-*nanos1* RNA, Figure 2), combinations of their respective BiFC fusions gave no significant signal above background. The same argument applies to VC-XveloSV plus VN-XveloSV, which obviously must be in the same particles. As a consequence of their large size, these RNP particles must accommodate many XveloSV molecules; this must be so in order to yield the strong fluorescence seen when GFP constructs are expressed. Similarly, there was no signal when self-interaction of another particle protein, Poc1B was tested. We conclude that the positive signals detailed in Figure 3 and Table 2 identified genuine inter-molecular interactions within germ plasm RNPs.

Examination of the protein structure of Xvelo1 gives little indication of its biological function. Both Xvelo isoforms contain a putative dynein light chain binding site, like the potential germ plasm protein Germes, which has been shown to interact with DLC-8 [21], but we find that this motif is not essential for the localisation of GFP-tagged XveloFL to germ plasm islands. However, in these experiments endogenously expressed Xvelo proteins are also present, so we cannot exclude the possibility that these interact with dynein and enable exogenous mutant Xvelo to localise via direct or indirect protein-protein interactions. The fact that XveloFL can interact with itself in BiFC experiments adds further credence to this possible scenario.

We attempted to establish a functional role for Xvelo by depleting its RNA, and hence the protein, in oocytes. In the case of XveloFL the protein was so stable that we were unsuccessful, and not surprisingly there was no germ plasm phenotype. On the other hand it proved possible to reduce XveloSV considerably, resulting in the consistent break-up of the germ plasm islands into smaller structures. This phenotype was produced both by an XveloSV-specific anti-sense oligo and one to the region common to both Xvelo transcripts. This suggests that Xvelo is important for the coherence of germ plasm islands. It is notable that mitochondria are poorly localised to germ plasm when XveloSV is depleted, so the effect is not limited to the RNP particles containing XveloSV protein. This in turn implies that the Hermes/Xvelo RNP particles are important for organising the coherence of germ plasm islands. Although the effect of XveloSV depletion is relatively subtle, it is quite possible that this is because of redundancy between it and XveloFL, which we cannot deplete.

While we have not measured endogenous RNAs it is very likely that they would behave in the same way as the injected RNA. We have previously argued that injected Cy5-nanos1 RNA exchanges into endogenous particles, as does YFP-Hermes [4]. The latter is co-extensive with all the endogenous Hermes particles revealed with antibody staining. If there were different endogenous RNA particles that did not behave like those containing Cy5-*nanos1* RNA, they could not contain Hermes, which is extremely unlikely.

The overall structure of microtubules in the germ plasm region appeared to be unaffected following XveloSV depletion. This indicates that XveloSV may mediate the assembly and maintenance of germ plasm via non-cytoskeletal components like nuage, or perhaps through interaction with microfilaments. Dissociating the latter with cytochalasin D tends to produce a small decrease in island size. This effect was consistently enhanced when the F-actin-binding domain of Utrophin was over-expressed (unpublished data).

From the depletion experiment it is possible that the decrease in island size results from loss of XveloSV RNA itself, rather than its encoded protein, but two facts argue against this. Firstly, in zebrafish only translatable Bucky ball mRNA can rescue eggs mutant for its gene [15] and there is every likelihood that the role of Bucky ball/Xvelo1 will be conserved. Secondly depleting XveloFL has no phenotype, even though the RNA, but not the protein, is destroyed. XveloFL mRNA contains all of the RNA sequence present in the XveloSV transcript. If the RNA has a direct role it is likely to be through binding protein or RNA, so the larger splice variant would be likely to bind all those targets bound by XveloSV. Because loss of XveloFL RNA has no effect, but that of XveloSV does, it is therefore likely that it is the SV protein product that is important.

Since only XveloFL protein is a component of the Balbiani body it is possible that the progression from this unitary structure to multiple cortical islands is dependent on XveloSV.

### Rbm24b and 42b have a potential role in germ plasm RNP structure

Rbm24b and 42b are the two other proteins from the yeast 2-hybrid screen that we have investigated in detail. They are RNA-binding proteins that are similar to Hermes in having a single RRM domain. GFP fusions of Rbm24b, and particularly Rbm42b, localise much more poorly into germ plasm than Hermes, XveloFL and XveloSV. However, BiFC experiments show that both proteins can associate very efficiently with Hermes, XveloFL and XveloSV in germ plasm RNP particles. Each multimerises with the other in these particles and Rbm24b interacts with itself there (Rbm42b self interaction was not tested). So long as the two Rbm proteins are highly expressed in oocytes, and this is likely since their mRNAs are relatively abundant, the two proteins are likely to be significant components of germ plasm particles.

Rbm24b has been found to be involved in mRNA metabolism in the muscle cells of *Xenopus* [34] (where it is also known as Seb4) and other vertebrates [35], [36]. One established role is in binding to the 3′-untranslated region of myogenin mRNA and enhancing its stability [37]. In muscles of *C. elegans* the Rbm24 homologue SUP-12 is involved in RNA splicing [38].

Rbm42b has been shown to interact with hnRNP K, which is involved in a variety of processes including RNA splicing, stability and translation [39]. Furthermore, Rbm42b was shown to bind to the 3′ UTR of p21 RNA and also to co-localise into stress granules with hnRNP K when cultured cells were subjected to stress. This is particularly interesting because there are parallels between the composition of stress granules and germ plasm [40], [41].

Our attempts to investigate whether Rbm24b and 42b have any role in germ plasm structure and behaviour was handicapped by the stability of the two proteins. We argued that although minor reductions in the levels of individual proteins might not have an observable effect, the synergistic effect of several simultaneous depletions could give rise to a phenotype. In our experiments depleting Rbm24b and 42b interfered with the localisation of exogenously introduced YFP-Hermes to germ plasm, possibly by reducing spare capacity to bind Hermes into germ plasm. When combined with the antisense depletion of XveloSV, the decrease in the size of germ plasm islands produced by the inclusion of the latter was somewhat augmented. In contrast, the ineffective XveloFL depletion has no effect on island size in combination with antisense Rbm24b and 42b. These experiments are not strong evidence as yet, but do provide a preliminary indication that these two RNA binding proteins have a role in germ plasm structure, specifically via Hermes recruitment.

### A pathway of RNA localisation?

Examination of the distribution of Hermes and its binding partners within the oocyte proved interesting, particularly when their mutual interactions were spatially mapped. While XveloFL was purely cytoplasmic, XveloSV, Hermes and the Rbm proteins were also intensely nuclear, as judged by the expression of their respective fusion proteins. On the other hand BiFC analysis revealed that Hermes only interacted with itself and with Rbm24b in the cytoplasm, where it also interacted with XveloFL and SV. In contrast, it interacted with Rbm42b both in the nucleus and the cytoplasm. Macroscopic RNP particles (∼1 µm) containing these proteins, as well as co-injected *nanos1* RNA, are not seen in the nucleus, but occur throughout the cytoplasm. A possible rationalisation of these results is that Hermes requires different RRM-containing partners for its function within distinct cellular compartments and that within the nucleus this role is fulfilled by Rbm42b. Clearly, something must be preventing the association of Hermes with itself and with Rbm24b in nuclei. This could be secondary modification or the presence or absence of another partner; a possibility for the latter is XveloFL, which is naturally confined to the cytoplasm. Adding an NLS to XveloFL to enable its transport into the nucleus could test this.

An attractive model to explain this multitude of interactions is that these proteins constitute the transient intermediary complexes of an RNA transport system between nucleus, cytoplasm and vegetal cortex, with the regional differences in composition reflecting the kind of RNP remodelling discussed by Lewis and Mowry [42]. In the case of XveloSV we were able to resolve large spherical structures in the nucleus of hemisected oocytes following the expression of its GFP fusion. Furthermore, these structures appeared to coincide with sites of interaction between XveloSV and Rbm42b or Hermes in oocytes sectioned following BiFC analysis. Although they are reminiscent of nuclear coiled or Cajal bodies [27], [28], which have been suggested to contain precursors of germ plasm [43], further work is needed to support this speculation.

It is notable that the cytoplasmic distribution of fluorescent particles between the nucleus and the cortex of the oocyte is similar whether one injects RNA alone or expresses different proteins in isolation or in combination (the protein distribution is most clearly resolved in BiFC experiments, presumably because the protein/protein interactions only occur within the particles, and thus there is no background fluorescence). This suggests that the molecules, which are localised by diffusion/entrapment, are entering pre-existing structures, rather than creating new entities in each case. This implies that there may be protein or RNA/protein complexes localised throughout the cytoplasm in a relatively static manner. Presumably these particles are anchored by the cytoskeleton and may originally have been transported to these positions by the cytoskeleton, but in the short term one can conceive of a transport mechanism by facilitated diffusion. If proteins are diffusing in and out of the particles, as previously demonstrated for Hermes [4] there could be net transport if the molecules are irreversibly tethered in the germ plasm at the periphery of the oocyte. Of course this process, if indeed it does occur, would be particularly relevant to localised RNAs, whereas proteins would need to remain throughout the pathway, rather than showing net transport. Further work is needed to test this model, to establish the roles of the proteins that we have identified, to find out how their interactions are controlled and to define other members of the transport pathway.

## Materials and Methods

### Ethics statement

This work was carried out under a UK-approved animal procedures project licence and approved by the University of Warwick Biological Ethics Committee.

### Yeast two-hybrid screening

In order to identify proteins interacting with the RNA-binding protein Hermes a yeast two-hybrid screen was performed, essentially as described by Hames et al [10] and references therein. Briefly the Hermes ORF was amplified using the primers 5′-AAAGAATTCATGAGCGGCATCAAGTCAGAC-3′ and 5′-AAAGAATTCTTAACAAAACTGCCGAGACTT-3′ from the plasmid pT7TS Hermes (kindly provided by Dr Paul Krieg), digested with EcoRI and ligated into the corresponding site of the LexA DNA binding domain - based bait plasmid pBTM116. For the two-hybrid screen, the yeast strain L40 was cotransformed with pBTM116-Hermes and a *X. laevis* oocyte cDNA library, constructed in the activation domain vector pVP16 (provided by Drs D. Kimelman and H. Farr) [17] by standard yeast techniques. Positive clones were selected on medium lacking leucine, tryptophan and histidine, in the presence of 15 mM 3-aminotriazole and checked with a filter assay for β-galactosidase activity. The inserts from 13 positive prey plasmids were then amplified using VP16 vector-specific primers 5′-GAGTTTGAGCAGATGTTA-3′ and 5′-TGTAAAACGACGGCCAGT-3′, which flank the NotI insertion site. Products were gel purified and directly sequenced with the primer 5′-GAGTTTGAGCAGATGTTA-3′. To confirm the specificity of interactions, the Hermes ORF was amplified using the primers 5′-AAAGGTACCCCATGAGCGGCATCAAGTCAG-3′ and 5′-AAAGGTACCTTAACAAAACTGCCGAGACTT-3′, digested with KpnI and ligated into the corresponding site within the activation domain vector pVP16 to generate pVP16-Hermes. Selected inserts from positive colonies (Table 1) were amplified and ligated into the bait plasmid pBTM116 and co-transformed with pVP16-Hermes into the yeast strain L40, then grown on selective media as described above.

#### In vitro translation and immunoprecipitation

Fusion proteins encoding eGFP-XveloFL, eGFP-XveloSV and HA-tagged Hermes [19]. were prepared in vitro using the TnT-coupled transcription/translation kit (Promega Corporation, Madison, USA). 1 µg of plasmid DNA was used in 50 µl reactions in the presence of ^35^S-methionine for individually translated proteins. When co-translation was performed 1 µg of DNA for each protein was included in the reaction together with appropriate RNA polymerases. One tenth of the translation products was resolved on 12% SDS PAGE gels and visualized by autoradiography.

For immunoprecipitation (IP) one fifth of the translation reaction was diluted to a final volume of 200 µl with IP buffer (150 mM NaCl, 50 mM Tris–HCl, pH 7.5, 1.5 mM MgCl_2_, 0.1% Triton X-100, 1X protease inhibitor cocktail (Roche)). Lysates were incubated at 4°C for 2–3 hours with rotation after the addition of 1 µg of mouse anti-HA (hemagglutinin) monoclonal antibody (H3663, Sigma). Following the addition of washed Protein-G agarose beads (40 µl) (Millipore), reaction mixtures were incubated overnight at 4°C with gentle mixing. After centrifugation pellets were washed four times in ice-cold IP buffer. Pellets were then resuspended in SDS–PAGE loading buffer and immunoprecipitated proteins were resolved on 12% SDS-PAGE gels, followed by autoradiography.

### GFP fusion constructs

The yeast two-hybrid screen led to the isolation of 13 positive clones (Table1) of which three, Xvelo1 (Long and short form), XRbm24b and XRbm42b were selected for further study. N-terminal eGFP fusions for these proteins were prepared by amplifying the eGFP coding sequence with the primers 5′-AAAAGATCTTAAATGGTGAGCAAGGGCGAGGAGCT-3′and 5′-AAAAAGCTTCTTGTACAGCTCGTCCATGCCGAGAG-3′. The product was digested with BglII and HindIII and ligated into the corresponding sites of pSPJC2L to prepare peGFPJC-N. The ORF for the long isoform of Xvelo1 (XveloFL) was amplified using the primers 5′-AAAAAGCTTATGAACACGACGGCACCTCCCCCAG-3′ and 5′-AAAGTCGACTTACAGTTTGCCATTCAGCTGCTTC-3′ using the IMAGE clone 9041603 (Source Bioscience Ltd, UK) as a template, digested with HindIII/HincII, and ligated into the corresponding sites of peGFPJC-N to prepare peGFP-XveloFL.

The ORF for the short isoform of Xvelo1 (XveloSV) was amplified using the primers 5′-AAAAAGCTTATGAACACGACGGCACCTCCCCCAG-3′ and 5′-AAAGTCGACCTACTTCTTCTGGATCAGTTCTAGT-3′, using Stage VI oocyte cDNA as a template, digested with HindIII/HincII, and ligated into the corresponding sites of peGFPJC-N to prepare peGFP-XveloSV.

peGFP-Rbm24b was prepared by amplifying the coding sequence with the primers 5′-AAAGTCGACATGCACACCACCCAGAAGGACACTA-3′and 5′-AAAGAATTCCTATTGCATGCGGTCAGCTTGCAGT-3′,using the IMAGE clone 5543457 as a template. The product was digested with HincII/EcoRI and ligated into the corresponding sites in peGFPJC-N.

The coding sequence for Rbm42b was amplified using the primers 5′-AAAGTCGACATGGCGGGGAAGAGCGGGGAGGAAA-3′ and 5′-AAAGAATTCCTATCTCAGGCCGAGCTTCTTCTTC-3′, using the IMAGE clone 8526901 as a template. The product was digested with SalI/EcoRI and ligated into the corresponding sites in peGFPJC-N to prepare peGFP-Rbm42b.

Sense strand mRNA for injection experiments was prepared by linearising the above constructs with XhoI and transcribing with SP6 RNA polymerase, using the Message Machine in vitro transcription kit (Ambion).

### Xvelo mutant constructs

N-terminally tagged GFP fusion constructs for Xvelo mutant proteins were prepared by PCR using the IMAGE clone 9041603 (XveloFL) as a template.

peGFPXveloFLΔ1 was prepared using the primers 5′AAAAAGCTTATGAACACGACGGCACCTCCCCCAG-3′ and 5′-AAAGTCGACTTAAGTTCTTCTCCCAGGACTGCTG-3′ to amplify the sequence encoding amino acid residues 1-114 of the long isoform of Xvelo1. peGFPXveloFLΔ2 was prepared using the primers 5′AAAAAGCTTATGAACACGACGGCACCTCCCCCAG-3′ and 5′-AAAGTCGACTTAAGAAGATCCTTCATCCCTAGAT-3′ to amplify the sequence encoding amino acid residues 1–451 of the long isoform of Xvelo1. cDNA sequences encoding amino acid residues 122–779 of the long isoform of Xvelo1 were amplified using the primers 5′-AAAAAGCTTGATCCACGGCAACAAGAATGTGCTT-3′ and 5′-AAAGTCGACTTACAGTTTGCCATTCAGCTGCTTC-3′ to construct peGFPXveloFLΔ3. peGFP-XveloSVΔ1 contained the c-terminal region of the short Xvelo isoform (XveloSV), specifically amino acid residues 205–269, this was prepared using the primers 5′-AAAAAGCTTGAAAAGCTCAAAAGAAAACCCCTGG-3′ and 5′-AAAGTCGACCTACTTCTTCTGGATCAGTTCTAGT-3′, using peGFP-XveloSV as a template. All inserts were digested with HindIII/HincII and ligated into the corresponding sites in peGFPJC-N. For in vitro RNA synthesis all mutant constructs were linearised with XhoI and transcribed with SP6 RNA polymerase, using the mMESSAGE mMACHINE kit (Ambion).

### Constructs for BiFC assays

The N-terminal and C-terminal halves of VENUS were amplified by PCR from the plasmids pCS2+VN154m9 and pCS2+VC155 (kindly provided by Prof. Jim Smith) respectively, and cloned into pSPJC2L. Venus fragments were fused to the N terminus of the ORFs encoding Hermes, XveloFL (long isoform), XveloSV (short isoform), XRbm24b, XRbm42b and xPix1(Poc1B) with a 10 amino acid flexible linker consisting of (Gly.Gly.Gly.Gly.Ser)_2_ inserted between the Venus and ORF fragments. This was done to ensure that the orientation/arrangement of the fusions in space was optimal to bring the Venus fragments into close proximity. The resultant VN-ORF/VC-ORF constructs in pSPJC2L were linearised by XhoI and transcribed in vitro using SP6 RNA polymerase. Potential protein-protein interactions within the germ plasm in vivo were assessed by injecting RNA encoding BiFC constructs into Stage VI oocytes and visualising by confocal microscopy after 48 hr in culture. Oocytes were also fixed with methanol/1% HCHO at −20°C over night following BiFC experiments and hemisected manually. Protein-protein interactions occurring in other subcellular compartments were detected using a stereo fluorescence microscope or by confocal microscopy.

### Antibody generation and affinity purification of Xvelo antisera

Polyclonal antibodies against XveloFL (long isoform) and XveloSV (short isoform) were raised in goats using the Eurogentec anti-peptide 28-day Speedy programme (Eurogentec, Seraing, Belgium). The antigens were synthetic peptides specific to XveloFL (H2N-QTREPRYETSKQGKQC-CONH2 and H2N-CKKNPKKSQSLSEPED-CONH2) and XveloSV (H2N-CSSLLKENKLELIQKK-COOH and H2N-CKIQPKSKSKISLQKR-CONH2). For the affinity purification of Xvelo-specific antibodies, sequences corresponding to nucleotides 864–1759 of the XveloFL cDNA and nucleotides 653–850 of the XveloSV cDNA [16], which contain the synthetic peptide sequences used for immunisation, were amplified by PCR and cloned into the bacterial expression vector pET14b to prepare pET14b-VFLΔ1 and pET14b-VSVΔ1 respectively. Fusion proteins were expressed in *E.coli* strain BL21(DE3), following the addition of 0.1 M IPTG. Pelleted cells were sonicated and proteins resolved by SDS PAGE. Resolved proteins were Western blotted onto nitrocellulose filters and stained with Ponceau S to visualise Xvelo proteins. Nitrocellulose bands corresponding to the Xvelo fusion proteins were then excised and destained. Total serum from final bleeds and preimmune serum was incubated with respective Xvelo fusion protein immobilised on nitrocellulose at 4°C overnight. Bound antibodies were eluted with 0.1 M acidic Glycine buffer (pH 2.5) and the pH adjusted to 7.0 by the addition of 1 M Tris pH 8.0. Sodium azide was then added to a final concentration of 5 mM and BSA to 1 mg/ml to stabilize the purified antibodies.

### Antisense depletion experiments

For antisense depletion experiments modified phosphorothioate oligonucleotides complementary to the RNA targets indicated below were synthesised. Stage VI oocytes were injected with 11 ng of each oligo, alone or in combination. XveloFL, 5′-A*T*C*C*TTAAGTCGGC*A*C*A*C-3; XveloSV, 5′-A*G*C*T*TTTCCTCCTC*T*T*T*C-3′ (specificity achieved by nine nucleotides flanking either side of the splice junction in Xvelo1 RNA [16]; XveloFL/SV, 5′-C*T*T*G*TTGCCGTGGA*T*C*A*G-3′ (this targets both the long and short forms of Xvelo RNA); Rbm24b, 5′-G*T*G*T*TGCAGCAGTA*A*G*T*G-3′; Rbm42b, 5′-A*A*G*C*GCGAGCAAGG*A*T*G*T-3′, where ‘*’ indicates phosphorothioate linkages (Invitrogen). Successful depletion of specific RNAs was assayed by semi-quantitative RT-PCR, essentially as described by Hudson et al. [44]. The primer sequences for each target sequence are given below, together with their respective amplification product, annealing temperature and the number of cycles used. For XveloFL; 5′-TGGTTCACCTGCTGCCATAC-3 and 5′-ATGGTTGGCGATTCTGCCTC-3′ (354bp, 51°C, 29 cycles). XveloSV; 5′-AAAAAGCTTGATCCACGGCAACAAGAATGTGCTT-3′ and 5′-AAAGTCGACCTACTTCTTCTGGATCAGTTCTAGT-3′ (447 bp, 51°C, 29 cycles). XveloFL/SV (amplifies both the long and short forms of Xvelo); 5′-AAAAAGCTTGAAAAGCTCAAAAGAAAACCCCTGG-3′ and 5′-AAAGTCGACCTACTTCTTCTGGATCAGTTCTAGT-3′ (197 bp, 51°C, 29 cycles).

Rbm24b; 5′-AAAGTCGACATGCACACCACCCAGAAGGACACTA-3′ (693 bp, 59°C, 31 cycles). Rbm42b; 5′-AAAGAATTCGGAACCGTCAGGATGGCGGGG-3′ and 5′-AAAGTCGACTCAGGGAAGTAGAGGTCCCAT-3′ (555 bp, 56°C, 29 cycles). ODC; 5′-GGAGCTGCAAGTTGGAGA-3′ and 5′-TCAGTTGCCAGTGTGGTC-3′ (131 bp, 55°C, 25 cycles).

### Oocyte culture, injection and microscopy

Oocytes were obtained, injected and examined by confocal microscopy as described previously [9], [10]. In these experiments oocytes were incubated in OCM medium within intact follicles (except for experiments where microtubules were stained with antibodies, where they were defolliculated with collagenase [45]). Endogenous XveloFL and XveloSV protein localisation experiments were performed on a fixed oocyte series (Stages I–VI) using isoform-specific, affinity purified goat polyclonals and a Rabbit Anti-Goat Dylight 594 (Jackson ImmunoResearch Laboratories, Inc) secondary antibody. Antibody staining was described by Machado et al. [9].
